# The Phenotypic and Transcriptomic Response of the *Caenorhabditis elegans* Nematode to Background and Below-Background Radiation Levels

**DOI:** 10.3389/fpubh.2020.581796

**Published:** 2020-10-16

**Authors:** Wayne A. Van Voorhies, Hugo A. Castillo, Cung N. Thawng, Geoffrey B. Smith

**Affiliations:** ^1^Molecular Biology Program and Biology Department, New Mexico State University, Las Cruces, NM, United States; ^2^Human Factors and Behavioral Neurobiology Department, Embry-Riddle Aeronautical University, Daytona Beach, FL, United States

**Keywords:** low radiation, biological response, *c. elegans*, deep biosphere, major sperm protein

## Abstract

Studies of the biological effects of low-level and below-background radiation are important in understanding the potential effects of radiation exposure in humans. To study this issue we exposed the nematode *Caenorhabditis elegans* to average background and below-background radiation levels. Two experiments were carried-out in the underground radiation biology laboratory at the Waste Isolation Pilot Plant (WIPP) in New Mexico USA. The first experiment used naïve nematodes with data collected within 1 week of being placed underground. The second experiment used worms that were incubated for 8 months underground at below background radiation levels. Nematode eggs were placed in two incubators, one at low radiation (ca.15.6 nGy/hr) and one supplemented with 2 kg of natural KCl (ca. 67.4 nGy/hr). Phenotypic variables measured were: (1) egg hatching success (2) body size from larval development to adulthood, (3) developmental time from egg to egg laying adult, and (4) egg laying rate of young adult worms. Transcriptome analysis was performed on the first experiment on 72 h old adult worms. Within 72 h of being underground, there was a trend of increased egg-laying rate in the below-background radiation treatment. This trend became statistically significant in the group of worms exposed to below-background radiation for 8 months. Worms raised for 8 months in these shielded conditions also had significantly faster growth rates during larval development. Transcriptome analyses of 72-h old naïve nematode RNA showed significant differential expression of genes coding for sperm-related proteins and collagen production. In the below-background radiation group, the genes for major sperm protein (*msp*, 42% of total genes) and sperm-related proteins (7.5%) represented 49.5% of the total genes significantly up-regulated, while the majority of down-regulated genes were collagen (*col*, 37%) or cuticle-related (28%) genes. RT-qPCR analysis of target genes confirmed transcriptomic data. These results demonstrate that exposure to below-background radiation rapidly induces phenotypic and transcriptomic changes in *C. elegans* within 72 h of being brought underground.

## Introduction

Life on earth has evolved with a constant exposure to ionizing radiation (1, 2). While all organisms are continuously exposed to ionizing radiation, natural rates of exposure can vary widely. Worldwide there is an ~1,000-fold range in natural background radiation levels, with radiation exposure levels ranging from under 50 nGy hr^−1^, to almost 30,000 nGy hr^−1^ (3, 4). No cytogenetic differences were documented in people living at elevated levels of radiation in Ramsar, Iran compared to control groups living at normal background (3). In a more thorough review of elevated radiation sites across the world (5), report little or no detrimental effects in residents of elevated radiation areas, but point out the need for more controlled studies. For the purposes of the discussion below, we follow (5) in defining normal background as levels ranging from 1 to 5 mSv/yr (114–570 nGy/hr).

Because of the known adverse effects of high levels of radiation exposure in biological systems, scientific and regulatory agencies have established occupational and public exposure limits. These are based on the “Linear No Threshold” (LNT) model. This model assumes that there is a linear increase in deleterious effects as radiation dose levels rise, and according to this model, no radiation level is considered safe (6). There are numerous animal model studies which contradict the LNT model and Skyes (7) has recently proposed that “Until those on one side of the debate can convince the other, it would be sensible to move forward toward a graded (risk-based) approach to regulation, where the stringency of control is commensurate with the risk, resulting hopefully in more sensible practical thresholds.”

While the high-dose region of this model is well-supported by epidemiological and experimental data, its validity at low doses, particularly near background values, has been challenged (8–10). Experiments have shown that cells grown at below normal levels of radiation exhibit potentially deleterious responses compared to cell grown under normal background radiation levels (11, 12). The biologic effects of below-normal background radiation are relatively unstudied (13). One reason for the lack of information on the effects of low radiation are the difficulties associated with conducting such studies. In order to obtain the low radiation levels required for such studies, experiments must be carried out deep underground to provide shielding from cosmic radiation or in lead-shielded incubators aboveground. Low-level, underground radiation experiments studies require that the geologic formation is itself a low radiation emitter with minimal levels of radon present.

The few studies that have been done in such sites show interesting biological responses in organisms grown in below-normal background radiation levels. Pioneering experiments examining effects of below-normal background radiation levels demonstrated that cultures of *Paramecium tetraurelia* grown at 11.4 nGy hr^−1^ had a reduced grow rate and longer generation time, compared to control cultures grown at 199 nGy hr^−1^ (14). A similar result was reported for bacterial cultures of *Synechococcus lividus* grown at 30 nGy hr^−1^ and 172 nGy hr^−1^. In both cases, normal growth was restored upon the addition of a radiation source equivalent to control levels (14).

In similar experiments Satta et al. reported that *Saccharomyces cerevisiae* cells grown at 4.5 nGy hr^−1^ exhibited a higher frequency of DNA damage when exposed to the genotoxin methyl-methane sulfonate compared to cultures grown at background levels (15). They also found numerous biological differences between the hamster tissue cell cultures grown at 4.5 nGy hr^1^ and normal background radiation levels. Specifically, they found that compared to cultures grown at normal background radiation levels, cells grown at low radiation levels had a lower cell density at confluence, increased accumulation of reactive oxygen species, an increased mutation rate when exposed to acute doses of gamma rays, and a higher rate of cellular apoptosis in the presence of cycloheximide (16). Castillo et al. (17–19) have documented a stress response in two bacterial species within 48 h of being brought underground and grown in the absence of normal radiation levels. Morciano et al. (20) were the first group to document these effects in a multi-cellular organism (*Drosophila)*, with reduced radiation causing a 30% reduction in fertility in males and females, while also causing an increase in life span.

Understanding the biological effects of low level radiation exposure has become more urgent in the face of the widespread and increasing use of ionizing radiation in medical imaging and treatments (21). Knowledge of the effects of low level radiation are also critical in determining appropriate levels of radiation exposure for workers involved with nuclear clean-up or other activities involving nuclear material. To further document the effects of low level radiation on a multicellular organism we conducted experiments at the U.S. underground nuclear waste repository at the Waste Isolation Pilot Plant (WIPP) located near Carlsbad, NM. For this study we grew parallel populations of the nematode *Caenorhabditis elegans* underground in an incubator that was shielded from radiation and in an incubator supplemented with KCl to give background radiation levels. We then compared basic life-history and gene expression patterns between these two groups to document if these low radiation levels induced any biological response.

## Materials and Methods

### Low Background Radiation Experiment, LBRE

All of the low level radiation experiments were carried out at a nuclear waste disposal site operated by the United States Department of Energy located 42 km east of Carlsbad, NM. This site, designated as the Waste Isolation Pilot Plant (WIPP), was designed to permanently store transuranic wastes generated from the U.S. military weapons program. The nuclear waste material is stored 650 m underground in a 610-m-thick Permian aged sea-salt deposit. The WIPP site began receiving nuclear waste in March 1999. An underground equipment fire and radiation release from a spontaneous fire in a waste storage drum in 2014 halted underground storage for ~3 years, with the site resuming emplacement of nuclear waste in January 2017.

In addition to nuclear waste storage, the WIPP site also has hosted several scientific projects that take advantage of the naturally occurring, extremely low background radiation levels found underground. These low background radiation levels are a consequence of the shielding from cosmic radiation provided by the underground depth of the site, and because the NaCl layer in which the site is located does not produce any natural radioactive decay particles. A low background radiation biology laboratory (the LBRE lab) was established in the North Experimental Area section of the mine in 2009 and is located ~one km from any nuclear waste. To further reduce background radiation levels, the LBRE laboratory houses a multi-ton, 2.5 × 2 × 2 m by 15 cm-thick steel vault that provides additional radiation shielding. This vault was made from steel produced prior to World War II and is free of any radioactive contamination caused by fallout from aboveground nuclear bomb testing.

Two Sable System (Sable Systems, Las Vegas, NV, USA) incubators were placed adjacent to each other in this vault. Because of this close spacing both incubators would have essentially identical air pressures. The control group was exposed to a radiation level of ~70 nGy hr^−1^, a level matching typical average surface background radiation levels. To provide this radiation level 2 kg of KCl was placed in four hollow plexiglass panels that surrounded the rack containing the Petri dishes in which the *C. elegans* were grown. KCl emits radiation due to the decay of the naturally occurring ^40^K isotope. By carefully regulating the amount and location of the KCl a radiation field approximating normal surface radiation can be created (17, 18). While a single radiation source will not fully represent the radiation spectrum of natural sources, as discussed previously (17), K is the dominant terrestrial radiation source and the majority of the photons from the ^40^K 1460 KeV emission undergo Compton scattering, which then produces a cascade of lower-energy electrons and deflected gamma rays. This results in a broad range of secondary ionization events with a wide spectrum of ionizing energies (22). Ion Chamber detector measurements were taken inside the KCl-supplemented incubator. The dose rate was measured to be 52 (+/- 8.7) nGy hr^−1^and, in combination with the dose received from the media (15.4 nGy/hr, see below), the KCl-supplemented cells were exposed to ~67.4 nGy/hr. Radon was measured to be 15.6 Bq/m^3^ in the underground (19) and is close to outdoor levels in the region, ~14.8 Bq/m^3^ (U.S. EPA map of radon zones) (23).

In order to shield radiation from the KCl control incubator, five water-filled 20-L carboys were placed around the outside of the incubator. The below background radiation *C. elegans* group was located in a separate incubator surrounded by an identical set of panels that were filled with 2 kg of NaCl. In previous work, radiation levels inside the vault were calculated to be ~0.16 nGy hr^−1^ by Monte Carlo MCNP analyses (17). However, due to the 25 mM potassium buffer present in the nematode growth medium, the below background radiation levels were substantial but too diffuse to measure and so were calculated: 15.4 nGy hr^−1^ from the NGM medium and 0.16 nGy/hr by MCNP to give ~15.6 nGy/hr for the dose rate for the shielded cells [the MCNP calculation was described by Castillo et al. (17)]. The incubators were set to 20.0°C, with temperatures monitored at 5-min increments with Hobo temperature data loggers (Pendant UA-002-64, Onset, Borne, MA, USA).

The phenotypic variables measured in these experiments were: (1) egg hatching success, (2) the body size of worms from larval development to adulthood, (3) developmental time from egg to egg laying adult, and (4) the egg laying rate of young adult worms. The advantage of measuring these four easy-to-quantify parameters is that they provide a very sensitive indicator of any factors influencing the physiological condition of the worm (24). Additionally, groups of worms were collected to determine patterns of gene expression at 24, 48, and 72-h time points by transcriptome analysis and RT-PCR verification.

A potential complication in measuring all of these variables, however, is that they are also very sensitive to the ambient temperature at which the worms are reared. For this reason, it is critical that the worms were maintained in incubators that are precisely able to regulate and maintain a constant temperature. To ensure that the temperature in the incubators remained within 0.1°C of the 20°C set-point we used Sable Systems incubators that employ Peltier-based heating and cooling units regulated by a proportional-integral-derivative temperature controller. Extensive testing of these incubators showed that all of the incubators were able to maintain the incubator temperature within 0.1°C of the set-point temperature over the course of the experiment. Additional details on this are provided in the Supplemental Materials.

### *C. elegans* Cultures

All experiments were done using the wild-type N2 strain obtained from the *Caenorhabditis* Genetics Center (Minneapolis, MN, USA). The worm culture used was revived from laboratory storage in liquid nitrogen in May 2015 and maintained in 15–20°C incubators prior to the start of the experiments. Worms were grown on Petri dishes filled with NGM [Nematode Growth Medium, (25)] inoculated with bacterial lawns of *Escherichia coli* strain OP50.

A starter culture of worms was prepared 3 days prior to the start of the experiment. Groups of around 25 adult worms laid eggs for 3 h to produce an age-synchronized cohort of eggs. The adults that developed from this age-synchronized cohort were then used to produce the eggs used in the experiment. To control for potential effects of parental age (26), the eggs used in all the experiments were laid by hermaphrodites that were between 72 and 78 h of age and the age of the starter (Po) worms was matched for each set of experiments.

One belowground experiment was done in December 2016, and a second in August 2017. The first experiment was started using age-synchronized 76-h-old Po worms that had developed in a 20°C incubator located at New Mexico State University. A temperature of 20°C is close to the thermal optimum for *C. elegans*, with the worms having maximal fecundity, rapid egg laying, and rapid development at this temperature (27). These worms were transported to the WIPP site in a portable incubator kept at 20°C and taken to the underground lab to start the experiment. Adult worms from these starter plates were placed on each of 18 NGM 65 mm Petri dishes for 3 h, with ~25 worms placed on each of the plates. At the end of this 3-h period the adult worms were removed from the plates and an individual egg was transferred onto a 35 mm NGM-filled Petri dish spotted with OP50 using a fine platinum wire. A total of 50–60 such plates were obtained; half of the plates were placed in the below background incubator, and the other half were placed in the KCl- supplemented control incubator. Additionally, nine 65 mm NGM plates with eggs were placed in each of the respective incubators. These plates were used over the course of the experiment to collect worms for gene expression studies. A subset of 125 eggs from the 65 mm Petri dishes was monitored for egg hatching success by observing how many eggs remained unhatched after 24 h. These groups of worms were then placed in the incubators and surrounded with plexiglass panels filled with KCl (surrogate for normal background radiation) or NaCl (low radiation group).

Development of these eggs to egg-laying adults was monitored for the next 72 h at ~24-h intervals. Each egg placed in the 35 mm Petri dish was individually measured for body based on an image of the worms recorded with a digital video camera (Scion, Fredrick, MD, USA) connected to a MS5 Lecia dissecting microscope and linked to an Apple MacBook Pro computer. These images were analyzed using ImageJ software to determine the length of the developing worms at 24, 48, and 72 h of age. Depending on the magnification, either a hemocytometer slide or a calibrated scale standard was used as a size standard and the worms were measured at magnifications between 40 and 80 X. The time of egg laying was ranked as age 0 h.

The rate of egg laying and age of first reproduction were determined by monitoring the developing worms at 2-h intervals once the worms were ~64-h old. The age of first reproduction was based on when the first laid egg was observed on the Petri dish. The egg-laying rate was then calculated from the number of eggs laid by the worm in the proceeding 2-h interval. If eggs were already present on the plate by the first observation interval, the age of first reproduction was calculated based on the average egg-laying rate of the group of worms. Due to safety restrictions, WIPP personnel had to be on the surface by early evening. Because of this worms could not be monitored for egg laying past the 74 h time point and it was not possible to collect data for the total number of progeny produced by a worm. If the worm had not laid eggs by the end of the day-three sampling period, it was not included in the analysis. Three such worms were removed from each of the background and low level radiation groups for the first underground experiment and one from each group for the second experiment.

The second underground low radiation experiment was done in August 2017. The intent of the second underground experiment was to see if long-term, multigenerational exposure to below background radiation levels had any detectable effect on the worms. While conceptually identical to the first experiment, the major difference between this experiment and the first was that the starter worms for the low radiation group had been exposed to below background radiation since December 2016, ~8 months. This worm culture was derived from the low radiation treatment group used in the December 2016 experiment and left in a 20°C incubator inside the steel vault. They were transferred to new plates four times prior to the start of second belowground experiment. When left on NGM plates for long periods *C. elegans* can complete approximately three generations before all the food on the plate is depleted. The remaining worms then reduce their metabolism and become relatively dormant, but quickly revive when placed on new NGM plates (28). Based on this, a conservative estimate is that the belowground worms would have been exposed to low level radiation conditions for a minimum of 10 generations. Other studies have shown that the effects of a biological perturbations to *C. elegans* can persist for many generations (29). The control group for the second underground experiment was derived from an identical population of *C. elegans* maintained and transferred in parallel in a 20°C surface incubator at New Mexico State University. To control for the potential effects of transporting the worms to the test site, the worms used to produce the eggs used in the experiment were reared for one full generation at the WIPP underground site while exposed to KCl radiation. This should minimize any residual effects of transport stress or acclimatization on the experimental results.

### RNA Extraction, Library Preparation, and Sequencing

Worms were washed off plates using ~2 ml of M9 buffer (25), and spun down at room temperature for 1 min at 2,000 rpm. The supernatant was removed and 400 ul of Trizol was added to lyse the worms, vortexed for 30 s and frozen at −80C. In both the initial short-term and the **second** long-term incubations, only the 72-h time-point yielded enough RNA for transcriptome analysis, and in the 2nd experiment this was further limited by only having **one** replicate control sample. Therefore, presentation of transcriptome data will be limited to only the **first** experiment.

One ug of RNA was used for cDNA library construction at Novogene (Sacramento, CA) using the NEBNext® Ultra 2 RNA Library Prep Kit for Illumina® (cat NEB **#E7775**, New England Biolabs, Ipswich, MA, USA) according to the manufacturer's protocol. After a series of terminal repair, poly-adenylation, and sequencing adaptor ligation, the double-stranded cDNA library was completed followed size selection and PCR enrichment. The resulting 250–350 bp insert libraries were quantified using a Qubit 2.0 fluorometer (Thermo Fisher Scientific, Waltham, MA, USA) and quantitative PCR. Size distribution was analyzed using an Agilent 2100 Bioanalyzer (Agilent Technologies, Santa Clara, CA, USA). Qualified libraries were sequenced on an Illumina Nova Seq 6000 Platform (Illumina, San Diego, CA, USA) using a paired-end 150 run (2 × 150 bases). ~20 million raw reads were generated from each library. An average of 26.5 ± 3.5 million reads were generated from each library.

### Experimental Design and Data Analysis

Two experiments were run in the WIPP underground, the first using naïve nematodes, the second using nematodes that were incubated for 8 months in sub-normal underground radiation conditions. For the phenotypic analyses of both experiments, there were between 15 and 30 biological replicates for each timepoint, represented by randomly chosen single nematodes on 15–30 NGM agar plates. It is standard practice in *C. elegans* studies to consider a single worm as an individual when analyzing data [e.g., (28, 30–33)]. Consistent with this, in our experiments a separate animal in an individual Petri dish was counted as a single biological replicate for the analysis of phenotypic traits. For the transcriptome analyses, only the 72-h timepoint from the 1^st^ experiment was analyzed. Two biological replicates (two independent NGM agar plates of ~ 300 nematodes on each plate) of the control (amended with KCl to represent background radiation) and treatment (below background radiation) underwent transcriptome pipeline analyses.

Statistical analysis of the phenotypic trait data was done using StatPlus v5 (AnalystSoft Inc, Walnut, CA). For the transcriptome analyses, three programs were utilized ArrayStar (ArrayStar® and QSeq®. Version 16.0.0. DNASTAR, Inc., Madison, Wisconsin, USA), CLC Genomics Workbench 12.2 (Qiagen Bioinformatics, Germantown, MD, USA) and Partek Flow (Partek Inc., St. Louis, MO, USA). The raw reads of RNA Seq were mapped against reference genome assembly of *C. elegans* (strain Bristol N2) (GCF_000002985.6) using analysis pipeline of Partek Flow software (Partek Inc., St. Louis, MO, USA) with default parameter. Alignment was performed with Bowtie 2 and differential gene expression was identified by Partek GSA algorithm. All RNA Seq data were screened for False Discovery Rate (FDR), and were accepted if FDR < 0.05 (34) with 2-fold change cut-off. Raw RNA sequences were trimmed, aligned and mapped against the reference genome of *C. elegans* (strain Bristol N2) (GCF_000002985.6). Expression of the genes was normalized by calculating RPKM (reads per kilobase of transcripts per million mapped reads]. The raw reads of RNA seq were also mapped against reference genome of *C. elegans* (strain Bristol N2) (NC_003279.8, NC_003280.10, NC_003281.10, NC_003282.8, NC_003283.11, NC_003284.9) using ArrayStar (ArrayStar® and QSeq®. Version 16.0.0. DNASTAR, Inc., Madison, Wisconsin, USA). The reads were normalized by RPKM method and gene expression were performed by using default parameter of ArrayStar. Raw RNA sequences were also analyzed by CLC genomics pipeline with default parameter. The raw RNA sequences obtained in this study were deposited at NCBI database (Accession number PRJNA631208). The significantly up and down regulated genes were analyzed for gene ontology (GO) term enrichment (FDR value < 0.05) using g:Profiler (35) and REVIGO (36) for visualization. Functional analysis was also performed by WormCat: an online tool for an annotation and visualization of C. *elegans* Genome scale data (37).

### RT-qPCR

The validity of differential expression was verified by using RT-qPCR for direct comparison with RNA Seq. The qPCR reactions (10 uL) were performed in triplicate using iTaq Universal One-Step RT-qPCR kit (BioRad, Hercules, CA, USA) with 0.5 μM of each primer (Supplementary Table 1), and 1 ng of total RNA as template. A first cDNA was synthesized by reverse transcription at 50°C for 10 min followed by RT inactivation at 95°C for 1 min. The reaction was directly followed by PCR amplification as follows: 40 cycles of denaturation: 30 s at 95°C; annealing: 30 s at 60°C; and extension: 30 s at 72°C. After amplification, the melt curve protocol followed with 30 s at 96°C and then 5 s each at 0.5°C increment between 60 and 95°C. The relative expression of the target genes *was* calculated using *act-1 and ubq-1* as reference genes and using the efficiency-corrected model (38). Ten genes from the transcriptome data were selected as potential reference gene for RT-PCR based on their downregulation (fold change < +1 and >−0.01). Primers were synthesized for 20 representative *msp* and *col* genes, and 10 reference genes using NCBI primer designing tool (https://www.ncbi.nlm.nih.gov/tools/primer-blast/index.cgi). From these 30 primer sets, properly calibrated standard RT-PCR curves were generated for 10 *msp*, 3 *col* and 8 reference genes. The act-1 and ubq-1 genes were chosen from a group of 10 potential reference genes (Supplementary Table 1) after being screened by NormFinder (39) and BestKeeper (40) method. For each comparison, 6 Ct values from two biological replicates were used for all calculations for relative expression of the target genes.

## Results

### Phenotypic Response to Below Background Radiation Levels

Conducting underground experiments at the WIPP site presented unusual challenges. The primary difficulty encountered was gaining access to the underground laboratory in a timely manner. Underground access was limited due to factors such as nuclear waste transportation issues, personnel access limitations due to ventilation restrictions in place since the radiation contamination event, planned and unplanned power outages, mine equipment failures, and above- and belowground safety drills. All of these factors restricted when data could be collected during the underground experiments. One experiment had to be terminated due to an underground power failure which caused the incubator temperatures to rise to unacceptable levels. As a result, complete phenotypic and partial genotypic data sets were obtained from two separate underground experiments.

The four phenotypic traits assayed: egg hatching success, body size over time, age at first reproduction, and early egg laying rate, proved to be sensitive markers for differences in radiation exposure. This was particularly the case for the population of *C. elegans* that had a long-term, multi-generational exposure to extremely low radiation levels.

#### Egg Hatch Rate, Body Size, and Life Cycle Time

For the second underground experiment, egg hatching rates were determined for 125 eggs divided into five sets of eggs for both the normal radiation and low below background radiation groups. Hatch rate was 100% for both groups (*n* = 125 for both groups).

There were no apparent differences between any of the groups in feeding or egg laying behavior. These observation periods, however, were minimized as it was important to keep the worms in the temperature and radiation controlled incubators for as much of the experiment as possible.

**Figure 1:**
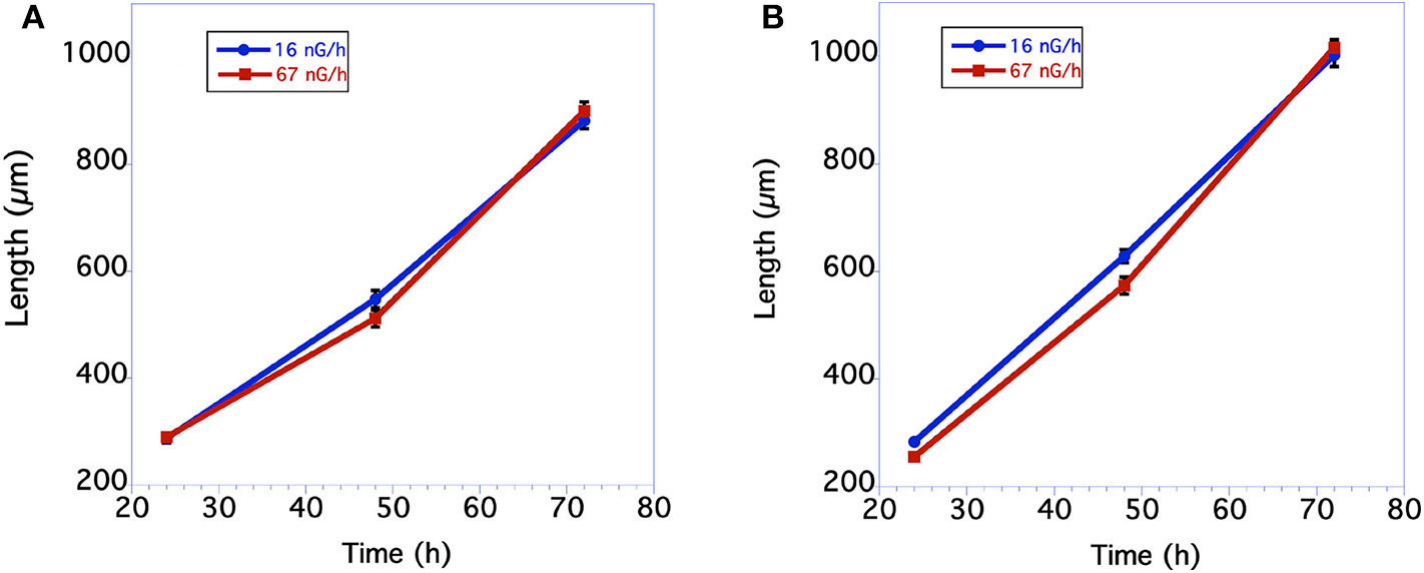
Comparison in length of *C. elegans* grown at normal background or low radiation levels. **(A)** Data from first underground experiment on 12/2016. The means and SEM are plotted for each group. *N* = 15–22 for each time point. No significant differences in worm size between the 2 groups at any of the 3 time points. For 24 h size *p* = 0.84 *T* = 0.21, df = 30; For 48 h size *p* = 0.12 *T* = 1.60, df = 38; for 72 h size *p* = 0.45 *T* = 0.76, df = 39. All data were analyzed using a Student's *T*-test with a two-tailed distribution. **(B)** Data from second underground experiment on 8/2017. The means and SEM are plotted for each group. *N* = 23–30 for each time point. Low radiation worms are significantly bigger at the 24 and 48 h time points. For 24 h size *p* = 0.00004 *T* = 4.55 df = 48; For 48 h size *p* = 0.008 *T* = 2.77 df = 54; for 72 h size *p* = 0.62 *T* = 0.50, df = 51. All data were analyzed using a Student's *T*-test with a two-tailed distribution.

**Figure 2:**
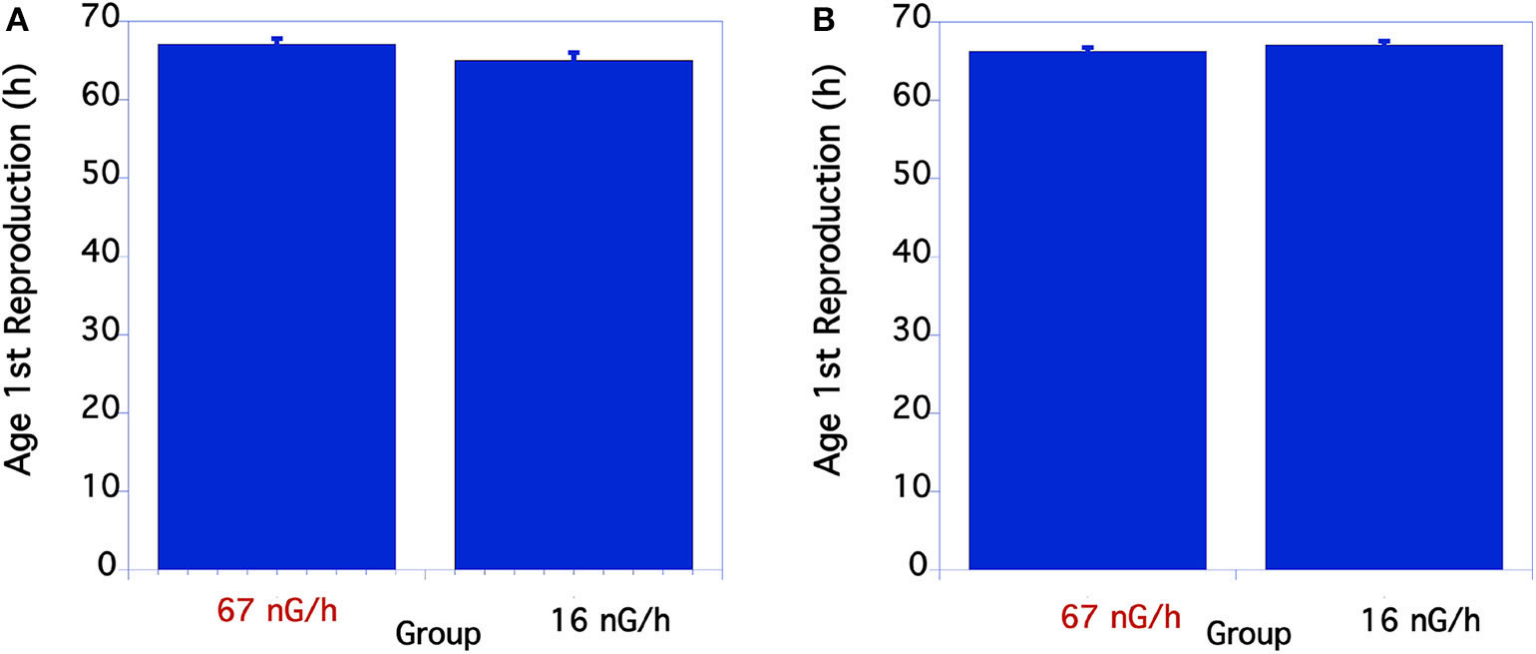
Comparison of time to complete life cycle *in C. elegans* grown at normal or low radiation levels. **(A)** Lifecycle time comparison of *C. elegans* in 12/2016. *N* = 18-22. *p* = 0.078 *T* = 1.8, df = 29. In the second underground experiment on 8/2017 the developmental times of the two groups was almost identical **(B)** *p* = 0.21 *T* = 1.26, df = 50. **(B)** From 8/17 Experiment. The means and SEM are plotted for each group. *N* = 23–30 for each time point. There were no significant differences in developmental time between the two groups.

Measurements of body size were based on comparisons of worm length. In nematodes there is a near perfect correlation with overall volume, i.e., size, and worm length. As seen in Figure 1A, in the first underground experiment the body size of the two groups was similar. The low level radiation group was slightly, but not statistically larger than the control level radiation group at 48 h of age. In the second experiment larvae growing in low radiation conditions for ~10 generations were significantly larger than larvae from the normal radiation level group through 48 h (Figure 1B). The adult body size of 72 h old worms from these two groups, however, was not significantly different. The time from when an egg was first laid until it developed into an egg-laying adult was compared between the normal and low level radiation groups. As seen in Figure 2A, in the first underground experiment the low radiation group developed slightly, but not significantly, faster than the Control radiation group. In the second experiment the developmental times of the two groups was almost identical (Figure 2B) and not significantly different. All data were analyzed using a Student's *T*-test with a two tailed distribution.

For the two aboveground experimental controls there were no significant differences between the life cycle time of the worms at any of the timepoints measured when compared within each experiment.

#### Egg Laying Rate

**Figure 3:**
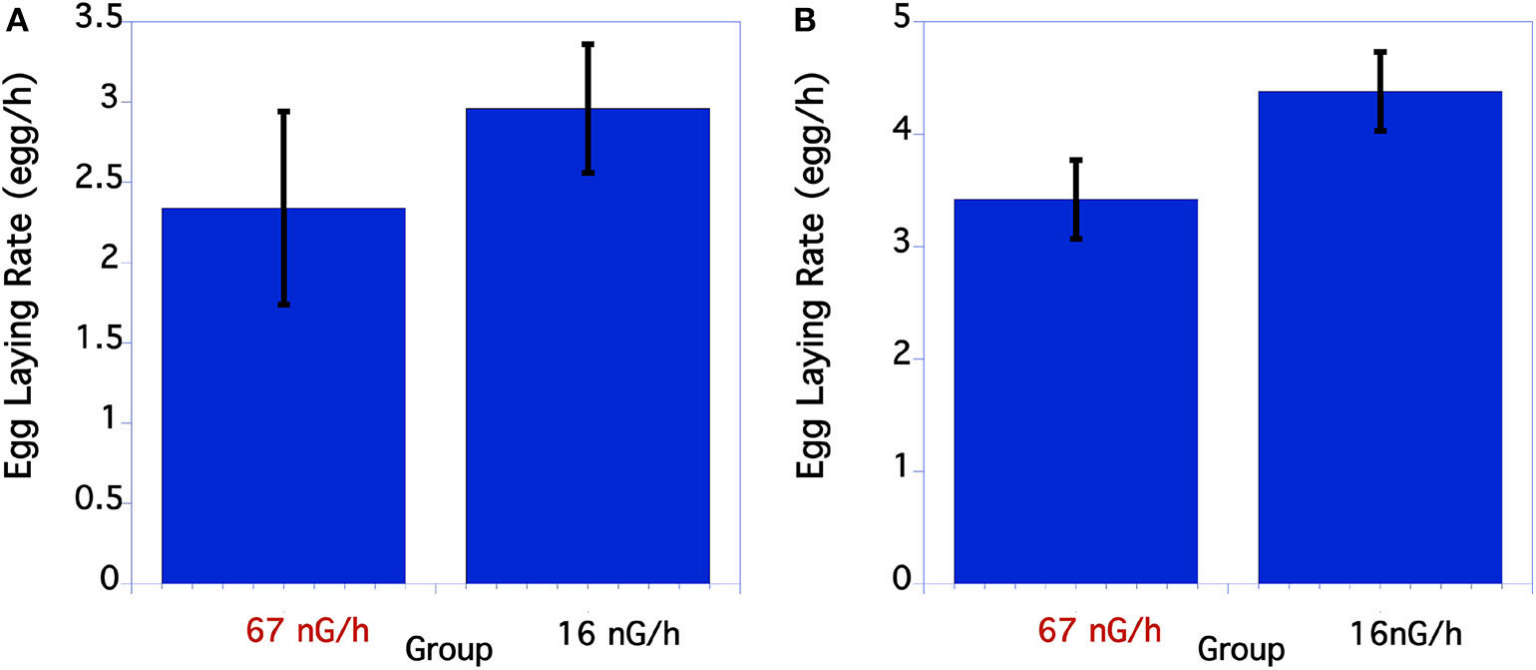
Comparison of egg laying rate of *C. elegans* grown at normal or low radiation levels. **(A)** Egg laying rate of *C. elegans* grown at high or low radiation levels 12/2016. There were no significant differences in egg laying rate. *P* = 0.39 *T* = 0.86, df = 29. **(B)** Data from second underground experiment on 8/2017. The means and SEM are plotted for each group. The lay rate of low radiation worms is significantly higher than the normal radiation group. *p* = 0.04 *T* = 2.10, df = 50.

The initial egg laying rate of *C. elegans* was compared between the control and low level, below background radiation groups. As seen in Figure 3A, in the first underground experiment the low-radiation group had a slightly, but not significantly greater egg-laying rate than the normal background radiation group. In the second experiment the egg-laying rate of the low radiation group was significantly greater than the normal radiation control group (Figure 3B).

For the two aboveground experimental controls there were no significant differences between the size of the worms at any of the timepoints measured when compared within each experiment.

#### Incubator Temperatures

Incubator temperatures remained very close to each other for all of the experiments (Supplementary Table 1). For the underground experiments the incubator temperatures were within 0.1°C of each other. If any biases existed from differences in temperature it would be that the normal radiation group would grow, develop, and lay eggs at a higher rate than the low radiation group, opposite to what was observed.

### Transcriptome Changes After Exposure to Below Background Radiation

**Figure 4:**
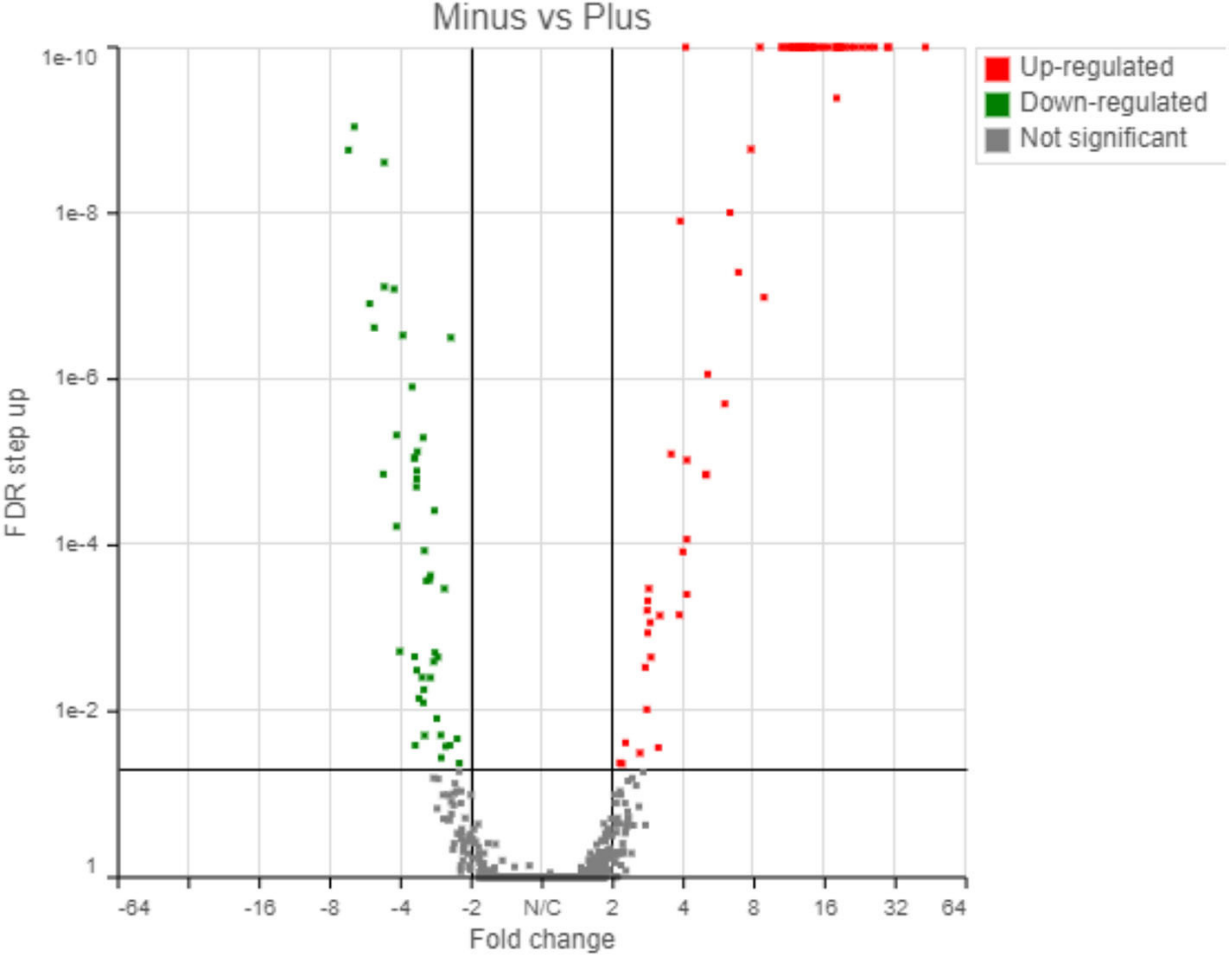
Transcriptome results from Partek analyses showing 67 genes upregulated and 46 genes downregulated in response to the radiation treatment (False Discovery Rate, FDR < 0.05).

The transcriptome analysis revealed a total of 5,053 genes detected using the Partek workflow software. Differential expression of control and treatment revealed that 67 and 46 genes were up- and down-regulated, respectively, based on a 2-fold change with a False Discovery Rate (FDR) < 0.05 (Supplementary Tables 2, 3). Using the Partek transcriptome analysis software (the other software packages will be compared below), volcano plot analysis of the 1st experiment indicated a statistically significant regulatory response to the radiation differences after only 72 h incubation in the two radiation treatments (Figure 4).

The up-regulated and down-regulated genes are shown in Supplementary Tables 2, 3. Interestingly, about half (49.5%) of the significantly up-regulated genes were different groups of major sperm proteins (*msp*, 42%), sperm-related genes (6%) and 1.5% were related to the major sperm genes. The rest of the upregulated genes were clustered in gene families related to collagen and cuticle related genes (16.4%), non-coding RNA (15%), and hypothetical proteins (7%). The most common down-regulated genes were collagen (*col*, 37% of total), cuticle related genes (28% of total) and hypothetical proteins (17.4%).

Similar transcriptional responses were demonstrated when three different transcriptome pipeline programs were used. For example, Table 1 shows the treatment to control ratios of all the up-regulated *msp* genes by Partek analysis and compares them to what was obtained by the two other RNA Seq analyses programs. All three programs gave similar results with only one exception (Table 1, marked in red) and, similarly, all the col genes identified as down-regulated by Partek were also identified as such by the other two programs (data not shown).

**Table 1:** Comparison of up-regulated fold-change values of the *msp* genes using three transcriptome pipelines.

**Gene**	**Partek**	**CLC genomic**	**DNA star**
*msp-78*	42.9	33.9	25.5
*msp-65*	29.5	39.6	40.9
*msp-10*	26.0	22.2	21.7
*msp-31*	24.5	ND	19.8
*msp-19*	22.9	16.2	22.6
*msp-59*	21.4	26.4	16.1
*msp-79*	20.5	18.7	13.9
*msp-113*	18.8	18.2	15.3
*msp-56*	18.2	29.1	19.4
*msp-81*	17.9	19.6	16.7
*msp-77*	17.8	30.1	24.3
*msp-53*	16.6	32.6	21.3
*msp-40*	15.8	25.4	20.1
*msp-49*	15.2	24.2	20.2
*msp-152*	14.0	20.2	23.9
*msp-57*	13.9	25.0	17.9
*msp-36*	13.1	23.8	17.4
*msp-55*	13.0	18.5	14.7
*msp-51*	12.8	14.7	12.9
*msp-76*	11.8	15.0	12.7
*msp-45*	11.7	−29.1	17.3
*msp-3*	11.4	20.2	17.3
*msp-33*	10.6	16.5	10.7
*msp-142*	10.5	28.4	22.0
*msd-4*	8.5	23.8	17.9
*msp-50*	6.9	19.7	16.7

*Fold-change values are from the ratio of treatment vs. control. ND, not detected*.

**Figure 5:**
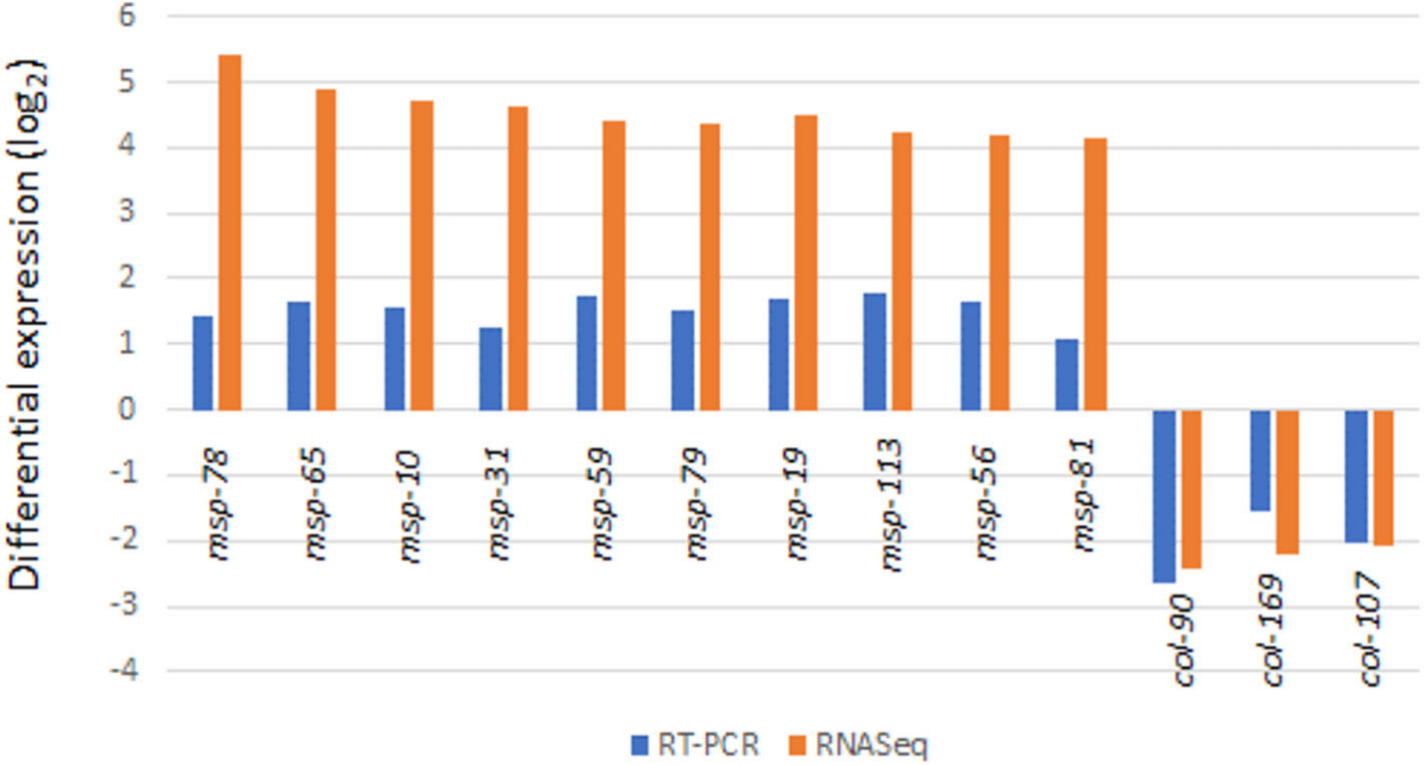
Validation of transcriptome results by RT-PCR analysis (for all values shown, *p* < 0.05).

Real-Time (RT) PCR was used to verify transcriptome results from 72 h culture (Figure 5). As expected, results of the 8 potential reference genes (*act-1, ubq-1, act-2, pmp-3, eif-3.C, tba-1, ama-1, rbd-1*) showed no difference in Cq values between the treatment and control RNA samples in agreement with transcriptome analysis results. By using two reference genes (*act-1* and *ubq-1*), 10 of the *msp* (*msp-78, msp-65, msp-10, msp-31, msp-59, msp-79, msp-19, msp-113, msp-56, msp-81*) and 3 of the *col* (*col-90, col-169, col-107*) genes gave similarly significant (*p* < 0.005) up and down RT-PCR responses of the RNA samples (Figure 5).

## Discussion

This study differs from most studies on the effects of low level radiation on organisms. Such studies typically expose a control group to normal background radiation levels and then compare this group to a group that is exposed to a group slightly higher radiation levels (41). In this study the effects of levels of a radiation exposure that was around 1/3 of normal background was compared to a group kept at normal background radiation levels. We hypothesized three outcomes this experiment. First, if any level of radiation has adverse biological effects, worms reared in below background radiation conditions should develop more quickly, lay eggs at a faster rate, and potentially grow faster than worms exposed to normal radiation levels. Conversely, if below background radiation levels have an adverse biologic effect, worms reared in these low radiation conditions should take longer to develop to adults, have a reduced rate of egg laying, and a slower growth rate compared to counterparts reared in normal background radiation levels. The null hypothesis is probably the most parsimonious, that is, the differences in radiation levels between the low level and normal background radiation are so minor that no physiological effect would be observed.

*C. elegans* is an ideal organism to use to investigate this question. It grows easily in culture, has a rapid life cycle and is one of the best-studied organisms in the world, with extensive knowledge available on its genetics and gene function, developmental biology, and physiology (42, 43). *C. elegans* has also become a useful model organism in toxicity assays. Studies in *C. elegans* have been validated as good predictors for the adverse effects of many chemicals in mammalian species (44).

In spite of the observations that *C. elegans* is both resistant to relatively high levels of radiation (45, 46) and also tolerant of oxidative stress (47), a significant phenotypic and transcriptomic response to these low levels of radiation was documented in this study. Worms in the below normal radiation environment had faster rates of larval growth, a faster rate of early egg laying, and more than 100 genes were differentially regulated, compared to normal background radiation levels.

From these results in *C. elegans*, there is no evidence of an obvious negative effect of depriving worms from normal levels of radiation. While an argument could be made that the observed increase in egg laying rate in the low radiation group is a stress response, such a response is the opposite to that usually seen when *C. elegans* is exposed to environmental stressors. When exposed to an array of environmental stresses such as reduced food levels, vibrations, temperature extremes, osmotically taxing environments, exposure to high radiation levels, moderate amounts of glucose, and hypoxia, *C elegans* typically both reduces its growth and egg laying rate (27, 28, 31–33, 48, 49). Early reproduction appears to be a critical factor in *C. elegans* ecology. Worms with a shorter generation time outcompete populations of mutant worms that produce more progeny but have a longer generation time (Hodgkins and Barnes (50). One factor which does increase the egg laying rate in a hermaphrodite *C. elegans* is when it mates with a male worm (51), an event which did not occur in these experiments.

In contrast, previous research has shown that bacteria and eukaryotic cell cultures exposed to below background levels of radiation exhibit a stress response compared to control groups exposed to normal surface levels of radiation (13, 14, 17, 19, 52, 53). These inhibitory effects on single-celled organisms may be considered a type of hormetic response in which background levels of radiation were needed for optimal growth (4, 54). Results here with the multicellular nematode did not show this response in that worms in reduced radiation had higher egg-laying rates than worms grown in the presence of KCl, a treatment designed to mimic background radiation levels.

Consistent with the *C. elegans* phenotypic response, there were more than 100 genes that were significantly regulated. This response was documented after only 72 h of the worms being brought underground with almost half of the significantly up-regulated genes in the low radiation treatment related to nematode sperm production. There are 85 distinct major sperm protein genes (MSP) located on all six of the nematode chromosomes (NCBI, https://www.ncbi.nlm.nih.gov/genome/?term=txid6239[orgn]), of which 28 were up-regulated in the below background radiation group. MSP was first identified as being involved in sperm motility and is required for the sperm's unusual amoeboid-like sperm “crawling” (55). More recent studies have found that MSP is also critical in other aspects of *C. elegans* reproduction including oocyte and egg development, ovulation, spermathecal valve dilation and parthenogenesis (51, 56–59). Recently, Maremonte et al. demonstrated that high radiation induced the down-regulation of 28 major sperm protein genes which was followed by a reduction in reproduction and the number of spermatids in *C*. *elegans* (60). Seeing how functionally multifaceted major sperm proteins are, it is likely that the upregulation of the *msp* genes is related to the increase in egg laying rate seen in the low radiation group.

Gene ontology analyses [REVIGO; (36)], listed oocyte development as one of the GO terms significantly upregulated and extracellular structure organization (i.e., cuticle development) as down regulated, and these terms are consistent with the most prevalent genes regulated. The fact that col genes were down regulated, but a smaller percentage was up regulated, is related to the fact that there are 186 distinct col genes in *C. elgans* (https://www.ncbi.nlm.nih.gov/genome/browse/#!/proteins/41/43998%7CCaenorhabditis%20elegans/col, NCBI) and they likely represent different functions. Using WormCat (37), the dominant upregulated genes were major sperm protein related to the reproductive system while the majority of downregulated genes are extracellular material related to cuticle development. Interestingly, three genes (*cpr-3, gst-22*, F17C11.11) which are known to be related to stress responses, were also downregulated.

In our previous work with two species of bacteria (*Shewanella oneidensis* and *Deinococcus radiodurans*), we have repeatedly documented within 48 h of bringing cells underground, a phenotypic and genotypic stress response to growing the cells in below background radiation (13, 17–19). Intriguingly, within 72 h of growth underground at WIPP, we have documented in *C. elegans* a similarly rapid biological response. The apparent inhibition in egg-laying rate of *C. elegans* in the presence of normal background levels of radiation (at least the gamma radiation provided by our underground KCl source) contrasts with the growth stimulation we've seen in some species of bacteria in background radiation. Nevertheless, in both model organisms, it is apparent that fitness costs and benefits are related to extremely small differences in levels of ionizing radiation. This minuscule stimulus and genome-wide response in prokaryotes and eukaryotes challenges our understanding of the biological role of normal, background levels of radiation.

As Lampe et al. (61) have pointed out, based on the physics of the two radiation fields of background and below background radiation, it is challenging to propose that biological organisms could sense these miniscule differences in ionizing radiation. This point was initially and reasonably brought up by Washington University's Jonathan Katz (62), and, in our response to his critique in which we agreed with his calculations, we posited the idea that biological “sensors” rival our most sensitive radiation detection instruments [Castillo and Smith response to Professor Katz, Castillo et al. (63)]. And, in agreement with the Morciano et al. (20) work with *Drosophila* underground at Italy's Gran Sasso lab, we provide further evidence that multicellular organisms are also capable of responding to these vanishingly low differences in radiation fields. These results and those coming from other deep underground laboratories provide impetus to document the mechanisms in single-celled prokaryotes and multi-celled eukaryotes of the response to these minute differences in ionizing radiation and to identify the organismal response apparatus. In our future work, we will continue to put paradigms in Physics (short-term, 1–100 nGy/hr gamma radiation cannot evoke a near-field response in biological targets), Biology (background and below background radiation effects are biologically irrelevant) and Policy (all radiation is deleterious in a Linear, No-Threshold manner) to the test.

## Data Availability Statement

The datasets presented in this study can be found in online repositories. The names of the repository/repositories and accession number(s) can be found in the article/Supplementary Material.

## Author Contributions

All authors listed have made a substantial, direct and intellectual contribution to the work, and approved it for publication.

## Conflict of Interest

The authors declare that the research was conducted in the absence of any commercial or financial relationships that could be construed as a potential conflict of interest.
