# Supplementary material for: The Phenotypic and Transcriptomic Response of the Caenorhabditis elegans Nematode to Background and Below-Background Radiation Levels
Source: Front Public Health. 2020 Oct 16;8:581796. doi: 10.3389/fpubh.2020.581796 (PMC7596186; doi:10.3389/fpubh.2020.581796)
Supplement: Supplementary file 1 [file Data_Sheet_1.docx]

Supplementary Material

**Supplemental Material: Temperature Control, Calibration and Measurement**

Most standard commercially available incubators would not meet the temperature requirements needed in this study, which requires that the incubators maintain a stable temperature within 0.1°C over a 72-hour period. For the underground portion of this study we used Sable Systems incubators that employ Peltier-based heating and cooling units regulated by a proportional-integral-derivative temperature controller. The aboveground control studies used Sable Systems incubators as well as small portable Peltier-effect based commercial refrigerators (Chefmate) modified with a high precision proportional temperature controller (Oven Industries, Mechanicsburg, PA, USA). Extensive testing of these incubators showed that all of the incubators were able to maintain the incubator temperature within 0.1°C of the set-point temperature over the course of the experiment. The temperature data loggers used in this study were calibrated in a water bath against a reference thermometer (Matheson 62109-10) that had been calibrated against a US National Bureau of Standards thermometer (NBS #1776857). The accuracy of the reference thermometer was ± 0.05°C. The Hobo temperature loggers used in the incubators read within <0.05°C of each other when tested in the calibration water bath. Based on this calibration, the temperature data recorded by these loggers were both accurate and precise.

For one of the surface control experiments the incubators differed in temperature by almost 0.2°C. For the surface experiments the relatively small difference in temperature were not reflected in any significant differences in the phenotypic traits assayed. Based on this it is unlikely that the slight (<0.1°C) difference between the low radiation and normal radiation groups would have been sufficient to cause differences in the traits measured.

**Supplementary Table 1.** Comparison of incubator temperatures used for 3 control experiments done at New Mexico State University and 2 sets of low radiation experiments done underground at the WIPP site near Carlsbad, NM. All temperatures were recorded using calibrated Hobo Pendant data loggers.

| Group | Date | Temp. | Se | N |
| --- | --- | --- | --- | --- |
| Low Rad | 12/16 | 19.78 | 0.002 | 1119 |
| Control | 12/16 | 19.86 | 0.006 | 1128 |
| Low Rad | 8/17 | 20.08 | 0.007 | 845 |
| Control | 8/17 | 20.15 | 0.009 | 885 |
| Surface | 3/18 | 20.35 | 0.017 | 252 |
| Surface | 3/18 | 20.16 | 0.008 | 264 |
| Surface | 4/18 | 20.18 | 0.014 | 887 |
| Surface | 4/18 | 20.08 | 0.015 | 887 |

**
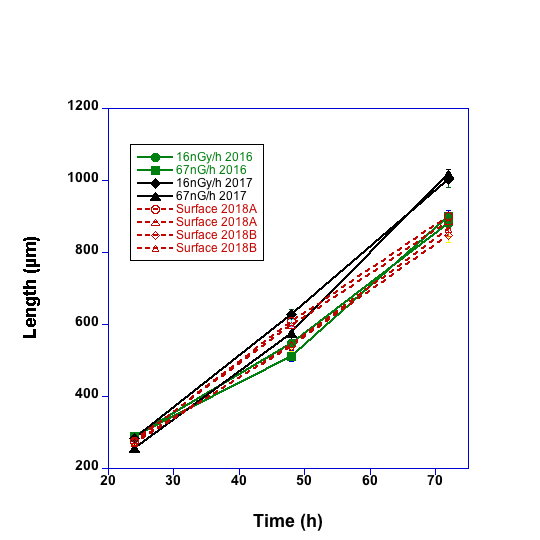
**

Figure 1. Comparison of *C. elegans* growth at high or low radiation levels (2 experiments) or on surface with normal background radiation (2 experiments).

**
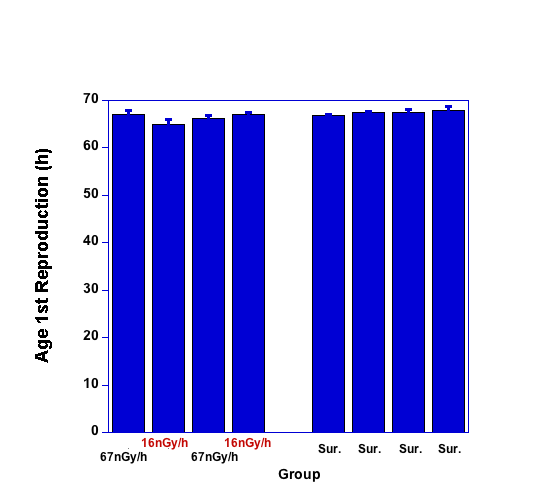
**

Figure 2. Comparison of lifecycle time of *C. elegans* grown at high or low radiation levels (2 experiments) or on surface with normal background radiation (2 experiments).


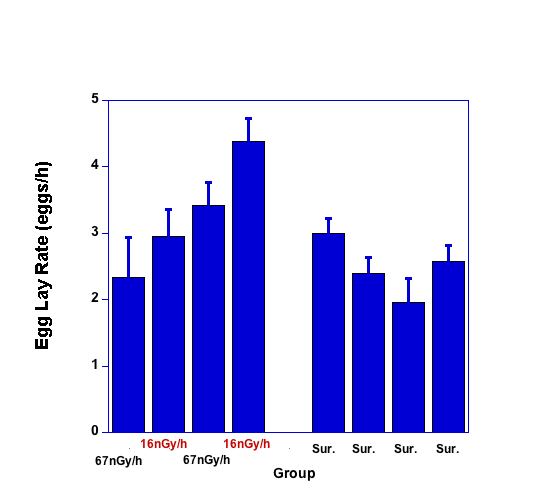


Figure 3. Comparison of egg laying rate of *C. elegans* grown at high or low radiation levels (2 experiments) or on surface with normal background radiation (2 experiments).

**
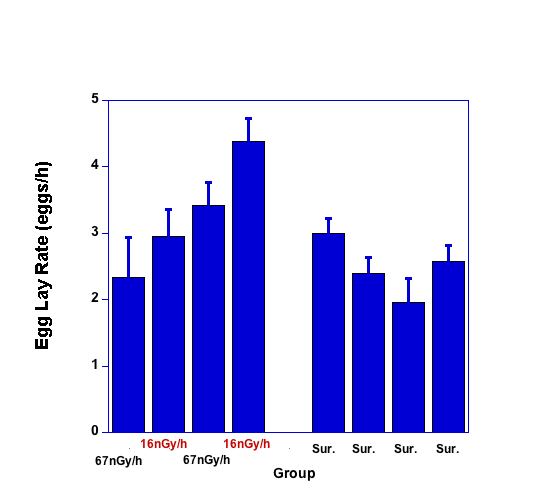
**

**Figure 4???**

**Supplementary Table 2.** Primers used in this study for qPCR. *^a^* Reference gene for gene expression normalization.

| **Gene** | **Function** | **Sequence (5' to 3')** | | **Product Size (bp)** | **Amplification Efficiency (%)** | **R^2^** | **Accession no.** |
| --- | --- | --- | --- | --- | --- | --- | --- |
|  |  | **Forward** | **Reverse** |  |  |  |  |
| *msp-78* | Major sperm protein | CATCGGCTCGTCGTATTGGA | GGCAAAAGCATCGCAGGAAA | 128 | 83.1 | 0.984 | NC_003282.8 |
| *msp-65* | Major sperm protein | CGTTCCACCAGGAGACATCC | ACTGCTTCCTTTGGGTCGAG | 195 | 82.7 | 0.990 | NM_068379.5 |
| *msp-10* | Major sperm protein | CATCGGCTCGTCGTATTGGA | GGCAAAAGCATCGCAGGAAA | 128 | 84.6 | 0.996 | NM_069359.6 |
| *msp-31* | Major sperm protein | CCATGTGGAGTTCTGGACCC | CATTCACGGCGGAATTGCTT | 152 | 87.7 | 0.997 | NM_062497.6 |
| *msp-59* | Major sperm protein | TCCTGTGATGCTTTCGCCTT | TAGCACAGGGCTCTATGGGT | 177 | 85.0 | 0.994 | NM_062497.6 |
| *msp-79* | Major sperm protein | CTCGACCCAAAGGAAGCTGT | CTGGAACCATTCACGACGGA | 147 | 86.8 | 0.994 | NM_069339.7 |
| *msp-19* | Major sperm protein | GCTTTCGCCTTTGGACAAGA | TAGCACAGGGCTCTATGGGT | 168 | 88.1 | 0.994 | NM_068359.6 |
| *msp-113* | Major sperm protein | CGTTCCACCAGGAGACATCC | ACTGCTTCCTTTGGGTCGAG | 195 | 91.7 | 0.996 | NM_068372.7 |
| *msp-56* | Major sperm protein | TGGATATGGTATCAAGACCACCAA | ACAGCTTCCTTTGGGTCGAG | 81 | 97.1 | 0.988 | NM_069361.6 |
| *msp-81* | Major sperm protein | CATCCAAACCCAACCGGGTA | CCAATACGACGAGCCGATGA | 102 | 89.7 | 0.998 | NM_069358.5 |
| *col-90* | Putative cuticle collagen | GGTGCTTCAATTTGTCTTCTTGGA | TTGCCACGTGTCATCAGCTA | 138 | 112.1 | 0.994 | NM_066328.6 |
| *col-169* | Col_cuticle_N domain- containing protein | GGCTACTTCCCTCAGACGTG | CGGGAACGGCAGAAATCAAC | 150 | 87.4 | 0.990 | NM_182455.7 |
| *col-107* | Col_cuticle_N domain- containing protein | GGAGCACAGCCAAACAACTG | TTCCGATTCCGTTTCCTCCG | 118 | 100.6 | 0.998 | NM_182305.4 |
| *act-1 ^a^* | Actin-1 | ACGACGAGTCCGGCCCATCC | GAAAGCTGGTGGTGACGATGGTT | 103 | 112.3 | 0.984 | NM_073418.6 |
| *Ubq-1^a^* | Ubiquitin-related | TCGAGGATGGACGCACTCTA | TCCGCGAAGACGAAGAACGA | 77 | 94.1 | 0.993 | NM_171868.7 |
|  |  |  |  |  |  |  |  |

**Supplementary Table 3.** Up-regulated genes

| **Gene** | **Description** | **FDR step up** | **Fold change** | **Log2** |
| --- | --- | --- | --- | --- |
| *msp-78* | major sperm protein | 4.95E-30 | 4.29E+01 | 5.421817067 |
| *msrp-2* | MS related protein | 1.00E-22 | 2.99E+01 | 4.156789136 |
| *msp-65* | major sperm protein | 2.71E-25 | 2.95E+01 | 4.613009502 |
| *msp-10* | major sperm protein | 1.41E-23 | 2.60E+01 | 4.417530079 |
| *msp-31* | major sperm protein | 2.41E-26 | 2.45E+01 | 4.880783862 |
| *msp-19* | major sperm protein | 2.71E-25 | 2.29E+01 | 4.515983818 |
| *msp-59* | major sperm protein | 3.81E-26 | 2.14E+01 | 3.602020765 |
| *msp-79* | major sperm protein | 3.54E-23 | 2.05E+01 | 4.235514535 |
| *Y59E9AR.1* | major sperm protein | 5.18E-21 | 1.93E+01 | 3.699138546 |
| *msp-113* | major sperm protein | 1.11E-24 | 1.88E+01 | 4.700173665 |
| *Y59E9AR.7* | major sperm protein | 1.72E-20 | 1.85E+01 | 4.357920455 |
| *msp-56* | major sperm protein | 5.29E-20 | 1.82E+01 | 3.6460554 |
| *ZK185.9* | non-coding RNA | 4.10E-10 | 1.79E+01 | 4.903868268 |
| *msp-81* | major sperm protein | 1.09E-22 | 1.79E+01 | 4.164813455 |
| *msp-77* | major sperm protein | 2.17E-26 | 1.78E+01 | 4.056434988 |
| *msp-53* | major sperm protein | 1.06E-21 | 1.66E+01 | 3.490044305 |
| *msp-40* | major sperm protein | 1.59E-20 | 1.58E+01 | 4.272934199 |
| *msp-49* | major sperm protein | 5.46E-15 | 1.52E+01 | 3.982876328 |
| *ssq-4* | sperm specific protein | 1.17E-13 | 1.45E+01 | 4.208554636 |
| *msp-152* | major sperm protein | 7.30E-18 | 1.40E+01 | 4.182199177 |
| *msp-57* | major sperm protein | 1.51E-18 | 1.39E+01 | 3.66916095 |
| *ssq-2* | sperm specific protein | 7.23E-14 | 1.39E+01 | 3.70795392 |
| *msp-36* | major sperm protein | 1.44E-18 | 1.31E+01 | 3.797523731 |
| *msp-55* | major sperm protein | 7.57E-12 | 1.30E+01 | 3.56180286 |
| *col-139* | col_cuticle_N domain-containing protein | 1.60E-24 | 1.30E+01 | 3.810817861 |
| *msp-51* | major sperm protein | 1.92E-17 | 1.28E+01 | 3.674848481 |
| *srpr-2.1* | non-coding RNA | 1.50E-19 | 1.27E+01 | 3.403796837 |
| *col-81* | col_cuticle_N domain-containing protein | 4.23E-23 | 1.25E+01 | 3.923622043 |
| *gipc-2* | PDZ-signaling protein | 2.87E-13 | 1.25E+01 | 3.547171908 |
| *srpr-2.2* | non-coding RNA | 4.08E-25 | 1.21E+01 | 3.797464763 |
| *msp-76* | major sperm protein | 2.32E-18 | 1.18E+01 | 3.856882134 |
| *msp-45* | major sperm protein | 6.71E-14 | 1.17E+01 | 3.641857907 |
| *msp-3* | major sperm protein | 1.77E-11 | 1.14E+01 | 3.702988863 |
| *col-129* | col_cuticle_N domain-containing protein | 2.23E-21 | 1.12E+01 | 3.50759979 |
| *msp-33* | major sperm protein | 2.12E-15 | 1.06E+01 | 3.080230632 |
| *msp-142* | major sperm protein | 5.90E-11 | 1.05E+01 | 2.029435347 |
| *ssq-1* | sperm specific protein | 1.03E-07 | 8.80E+00 | 3.38773844 |
| *msd-4* | major sperm protein | 1.88E-11 | 8.46E+00 | 4.165485881 |
| *F08H9.2* | hypothetical protein | 1.70E-09 | 7.75E+00 | 2.953520317 |
| *msp-50* | major sperm protein | 5.14E-08 | 6.85E+00 | 2.65704148 |
| *col-133* | col_cuticle_N domain-containing protein | 9.84E-09 | 6.31E+00 | 1.952967109 |
| *bli-6* | col-cuticle-N-domain protein | 1.98E-06 | 5.99E+00 | 2.77574884 |
| *---* | RNA | 8.68E-07 | 5.07E+00 | 3.137670871 |
| *rrn-1.2* | RNA | 1.39E-05 | 4.99E+00 | 2.341412832 |
| *rrn-1.1* | RNA | 1.42E-05 | 4.97E+00 | 2.582182915 |
| *col-62* | col-cuticle-N-domain protein | 9.39E-06 | 4.13E+00 | 1.823932252 |
| *rrn-3.1* | RNA | 3.89E-04 | 4.13E+00 | 2.046276057 |
| *rrn-3.56* | RNA | 8.47E-05 | 4.13E+00 | 2.319182187 |
| *rpl-30* | ribosomal protein | 3.10E-11 | 4.08E+00 | 2.314676062 |
| *ssp-10* | sperm specific protein | 1.20E-04 | 3.97E+00 | 2.044852443 |
| *col-7* | cuticle collagen 7 | 1.24E-08 | 3.87E+00 | 1.989069711 |
| *---* | RNA | 6.88E-04 | 3.84E+00 | 1.507327042 |
| *col-159* | col-cuticle-N-domain protein | 7.94E-06 | 3.54E+00 | 2.044961902 |
| *col-124* | col-cuticle-N-domain protein | 7.00E-04 | 3.17E+00 | 1.493543586 |
| *C34B2.8* | hypothetical protein | 2.72E-02 | 3.12E+00 | 1.485435056 |
| *F23A7.8* | hypothetical protein | 2.22E-03 | 2.90E+00 | 1.940560565 |
| *T05G5.17* | non-coding RNA | 8.50E-04 | 2.88E+00 | 1.663745667 |
| *rps-14* | ribosomal protein | 3.32E-04 | 2.84E+00 | 1.52786536 |
| *K08D12.6* | hypothetical protein | 4.65E-04 | 2.82E+00 | 1.492690655 |
| *F23A7.4* | hypothetical protein | 1.13E-03 | 2.81E+00 | 1.53766069 |
| *col-142* | col-cuticle-N-domain protein | 6.07E-04 | 2.80E+00 | 1.456786978 |
| *C35D10.17* | mitochondrial protein | 9.48E-03 | 2.79E+00 | 1.479508519 |
| *his-56* | histone H4 | 2.95E-03 | 2.74E+00 | 1.178101487 |
| *hpo-8* | CoA dehydratase | 3.17E-02 | 2.61E+00 | 1.64228485 |
| *col-92* | col-cuticle-N-domain protein | 2.38E-02 | 2.26E+00 | 1.383112307 |
| *nlp-24* | neuropeptide like protein | 4.28E-02 | 2.19E+00 | 1.097128991 |
| *rps-13* | ribosomal protein | 4.16E-02 | 2.14E+00 | 1.128642059 |

**Supplementary Table 4.** Down-regulated genes

| **Gene** | **Description** | **FDR step up** | **Fold change** | **Log2** |
| --- | --- | --- | --- | --- |
| *rol-8* | cuticle collagen 6 | 1.74E-09 | -6.69E+00 | -2.65987 |
| *col-41* | col-cuticle-N-domain protein | 9.10E-10 | -6.32E+00 | -2.74142 |
| *col-90* | Putative cuticle collagen 90 | 1.23E-07 | -5.44E+00 | -2.23612 |
| *sqt-2* | col-cuticle-N-domain protein | 2.39E-07 | -5.20E+00 | -2.23563 |
| *rol-6* | cuticle collagen rol-6 | 1.39E-05 | -4.75E+00 | -2.09664 |
| *col-17* | collagen | 2.45E-09 | -4.71E+00 | -2.44245 |
| *col-169* | col-cuticle-N-domain protein | 7.69E-08 | -4.71E+00 | -2.37784 |
| *col-107* | col-cuticle-N-domain protein | 8.19E-08 | -4.28E+00 | -1.97289 |
| *cpr-3* | proteinase | 5.92E-05 | -4.18E+00 | -1.29281 |
| *sqt-1* | cuticle collagen sqt-1 | 4.70E-06 | -4.18E+00 | -1.84135 |
| *pqn-32* | DB domain | 1.89E-03 | -4.05E+00 | -2.06197 |
| *col-167* | col-cuticle-N-domain protein | 2.96E-07 | -3.93E+00 | -1.68532 |
| *dpy-5* | cuticle collagen dpy-5 | 1.23E-06 | -3.58E+00 | -1.76802 |
| *col-3* | col-cuticle-N-domain protein | 8.87E-06 | -3.51E+00 | -1.81181 |
| *col-65* | col-cuticle-N-domain protein | 2.19E-03 | -3.50E+00 | -1.80229 |
| *col-10* | cuticle collagen 10 | 8.89E-06 | -3.49E+00 | -1.7752 |
| *dpy-2* | cuticle collagen dpy-2 | 2.56E-02 | -3.48E+00 | -2.2494 |
| *col-168* | col-cuticle-N-domain protein | 1.97E-05 | -3.44E+00 | -1.77711 |
| *col-117* | col-cuticle-N-domain protein | 1.58E-05 | -3.43E+00 | -1.78113 |
| *F53B2.8* | hypothetical protein | 3.19E-03 | -3.43E+00 | -1.5262 |
| *col-144* | col-cuticle-N-domain protein | 1.27E-05 | -3.42E+00 | -2.06284 |
| *dpy-13* | cuticle collagen dpy-13 | 7.44E-06 | -3.41E+00 | -1.66789 |
| *lpr-3* | lipocalin related protein | 6.97E-03 | -3.35E+00 | -1.58127 |
| *dpy-3* | col-cuticle-N-domain protein | 3.89E-03 | -3.24E+00 | -1.59609 |
| *col-125* | col-cuticle-N-domain protein | 5.04E-06 | -3.22E+00 | -1.63952 |
| *abu-14* | protein response | 7.84E-03 | -3.21E+00 | -1.38481 |
| *gst-22* | Glutathione S-Transferase | 5.46E-03 | -3.19E+00 | -2.01616 |
| *Y47D7A.13* | hypothetical protein | 1.16E-04 | -3.18E+00 | -1.51774 |
| *mlt-11* | hypothetical protein | 1.95E-02 | -3.17E+00 | -1.80625 |
| *ZK180.5* | hypothetical protein | 2.68E-04 | -3.12E+00 | -1.47761 |
| *cut-2* | cuticlin-2 | 2.61E-04 | -3.02E+00 | -1.53472 |
| *ram-2* | col-cuticle-N-domain protein | 2.31E-04 | -2.99E+00 | -1.77645 |
| *F41F3.3* | hypothetical protein | 3.92E-03 | -2.99E+00 | -1.69821 |
| *tts-2* | non-coding RNA | 2.52E-03 | -2.90E+00 | -1.58032 |
| *dpy-4* | col-cuticle-N-domain protein | 3.82E-05 | -2.88E+00 | -1.67581 |
| *col-170* | col-cuticle-N-domain protein | 1.96E-03 | -2.86E+00 | -1.74536 |
| *F17C11.11* | hypothetical protein | 1.22E-02 | -2.81E+00 | -1.6844 |
| *F54E2.1* | hypothetical protein | 2.22E-03 | -2.78E+00 | -1.49287 |
| *dpy-7* | cuticle collagen dpy-7 | 1.93E-02 | -2.70E+00 | -1.43518 |
| *cebp-1* | enhancer binding protein | 3.62E-02 | -2.69E+00 | -1.66544 |
| *K02E11.10* | hypothetical protein | 3.32E-04 | -2.61E+00 | -1.20441 |
| *col-130* | col-cuticle-N-domain protein | 2.63E-02 | -2.58E+00 | -1.79764 |
| *sqt-3* | cuticle collagen 1 | 2.56E-02 | -2.47E+00 | -1.30736 |
| *col-73* | col-cuticle-N-domain protein | 3.15E-07 | -2.45E+00 | -1.3667 |
| *col-166* | col-cuticle-N-domain protein | 2.14E-02 | -2.30E+00 | -1.42958 |
| *sqst-1* | SeQueSTosome related | 4.19E-02 | -2.26E+00 | -1.17358 |
